# Deep-Neural-Network-Aided Genetic Association Testing in Samples with Related Individuals

**DOI:** 10.3390/cimb48030273

**Published:** 2026-03-04

**Authors:** Xiaowei Wu

**Affiliations:** Department of Statistics, Virginia Tech, 250 Drillfield Drive, Blacksburg, VA 24061, USA; xwwu@vt.edu

**Keywords:** genome-wide association studies, machine learning, deep neural network, deep learning, ensemble learning, related individuals

## Abstract

Genome-wide association studies (GWAS) have successfully identified thousands of genetic loci associated with complex traits and diseases, providing critical insights into genetic architecture, biological pathways, and disease mechanisms. With the advance of machine learning, the analytical scope of GWAS can be substantially expanded by enabling joint modeling, nonlinear effects, and integrative analysis. However, deep learning approaches remain underutilized in augmenting traditional GWAS frameworks, particularly in the presence of cryptic relatedness among sampled individuals. In this paper, we propose a deep neural network (DNN)-based machine learning method to assist genetic association testing in samples with related individuals. By approximating the phenotype–genotype relationships in classical association tests and combining approximations across multiple tests, the proposed method aims to improve predictive performance in the identification of associated variants. Simulation studies demonstrate that our approach effectively complements conventional statistical methods and generally achieves increased power for detecting genetic associations. We further apply the method to data from the Framingham Heart Study, illustrating how DNN-based machine learning can facilitate the identification of genome-wide SNPs associated with average systolic blood pressure.

## 1. Introduction

Over the past two decades, genome-wide association studies (GWAS) have achieved remarkable success. Leveraging increasingly sophisticated statistical methodologies, GWAS have identified thousands of genetic variants underlying complex human diseases [1]. These discoveries have substantially advanced our understanding of disease biology and have driven progress in predictive genomics and precision health.

Classical GWAS relies primarily on statistical modeling, with the fundamental goal of scanning millions of single-nucleotide polymorphisms (SNPs) across the genome in large cohorts and testing each variant for associations with phenotypic variation. A wide range of association testing methods has been developed to accommodate different study designs and trait types. For example, logistic or linear regression models are commonly used to test marginal effects of individual variants [2]. When major confounders such as population stratification or cryptic relatedness are present, linear mixed models (LMMs) are often employed [3]. For longitudinally measured traits, appropriate approaches include varying coefficient models (VCMs), generalized estimating equations (GEE), and functional data analysis (FDA) methods [4,5,6]. In the context of rare variant and sequencing-based GWAS, burden tests and sequence kernel association tests (SKAT) have been proposed [7,8]. Despite the central role of statistical modeling in GWAS and its continued status as the gold standard for variant discovery, these approaches are often limited in their ability to capture joint genetic effects, nonlinear relationships, and complex interactions among variants [9].

Driven by rapid growth in data availability and advances in computational power, machine learning (ML), particularly deep learning (DL), has experienced explosive development in recent years. ML and DL have emerged as powerful and efficient tools for extracting meaningful patterns from complex, large-scale, and high-dimensional data. These state-of-the-art methods are increasingly being applied in GWAS to complement traditional statistical modeling. Typical applications include polygenic risk prediction [10], variant prioritization [11], epistasis detection [12], multi-omics integration [13], and functional effect prediction [14]. In contrast to classical statistical modeling, ML and DL approaches are generally not grounded in formal inferential frameworks. As a result, they can be more flexible and potentially more powerful for detecting atypical GWAS signals, such as nonlinear effects, ultra-rare variant effects, or effects obscured by incomplete or low-quality data, although they often suffer from limited interpretability and stringent sample size requirements [15,16]. Despite their rapid adoption, the full potential of ML and DL methods remains underexplored, particularly in GWAS settings with cryptic relatedness among sampled individuals.

In this paper, we investigate a deep learning approach for genetic association testing in samples containing related individuals. Through extensive simulation studies, we demonstrate that (1) DL models can effectively emulate classical association tests, achieving comparable or improved statistical power while maintaining proper control of type I errors; (2) informative features, such as allele frequency estimates and other GWAS summary statistics, can be leveraged to reduce the dimensionality of the DL inputs, resulting in comparable but more computationally efficient predictive performance; and (3) emulations of multiple association tests can be integrated through ensemble learning to enhance the detection of association signals. We further apply the proposed deep-neural-network-based test to a whole-genome analysis of average systolic blood pressure measurements from the Framingham Heart Study.

## 2. Materials and Methods

### 2.1. Statistical Modeling Approach for Association Testing in Samples with Related Individuals

In a genetic association study involving a group of n individuals, suppose that data are collected for a phenotypic trait Y (hereinafter we focus on quantitative traits), several covariates (arranged as columns of a design matrix W, with intercept included), and genotype X of an SNP variant of interest. The typed SNP is assumed to be biallelic with alleles arbitrarily labeled “0” and “1”, and the ith component of vector $X,\;X_{i}\in \{0,\;1,\;2\}$ is obtained by counting the number of alleles of type 1 in individual i, for 1 ≤ i ≤ n. The relatedness of these sampled individuals is assumed to be known and can be described by the following kinship matrix:

(1) $$\Phi =\left(\begin{array}{cccc} 1+h_{1} & 2\phi_{12} & \ldots & 2\phi_{1n}\\ 2\phi_{12} & 1+h_{2} & \ldots & 2\phi_{2n}\\ \vdots & \vdots & \ddots & \vdots \\ 2\phi_{1n} & 2\phi_{2n} & \ldots & 1+h_{n} \end{array}\right)$$

where $h_{i}$ is the inbreeding coefficient of individual i and $\phi_{ij}$ is the kinship coefficient between individuals i and j, 1≤ i, j≤ n. For outbred individuals, Φ represents the correlation matrix of individual genotypes. If the sampled individuals come from multiple families, the corresponding kinship matrix is block-diagonal, where each diagonal block represents the kinship matrix of a family and the off-diagonal blocks are **0** matrices.

Statistical methods for testing associations in samples with related individuals generally fall into two categories: the prospective test and the retrospective test. In the prospective test, the trait Y is treated as random and is characterized by the following linear mixed model:

(2) $$Y|(W,X)∼N(W\beta +X\alpha ,\Sigma ),\;\Sigma =\sigma_{e}^{2}I+\sigma_{a}^{2}\Phi$$

where I is the identity matrix and $\sigma_{e}^{2},\sigma_{a}^{2}$ are two variance components attributed to the random measurement error and the additive polygenic random effects, respectively. Based on this model, different statistical tests, e.g., t test, score test, or likelihood ratio test, can be developed to test α=0. The retrospective test, on the other hand, treats the genotype X as random. Under Mendelian inheritance, the genotype X can be modeled by a binomial random vector with dependent components, and, as such, based on Y and W, its mean and covariance can be seen as

(3) E[X|Y,W]=2p⋅1+γΦR

and

(4) $$Cov(X|Y,W)=\sigma_{X}^{2}\Phi$$

where $R={\hat{\Sigma }}_{0}^{-1}(Y-W{\hat{\beta }}_{0})$ is the transformed phenotypic residual, obtained from the trait model under the null hypothesis of no genetic association: $Y=W\beta_{0}+\epsilon$ and $\epsilon ∼N(0,\Sigma_{0})$. In this null model, $\beta_{0}$ represents the regression effect of the covariates, and $\Sigma_{0}=\sigma_{e}^{2}I+\sigma_{a}^{2}\Phi$ is the trait covariance matrix. In the above formulas, p is the allele frequency of the SNP variant, and $\sigma_{X}^{2}=2p(1-p)$ is the marginal variance of X. Using this setting, testing γ=0 is usually accomplished using a quasi-likelihood score test [17].

For the prospective likelihood score test, the test statistic (denoted by $S_{LS}$) is derived as

(5) $$\begin{array}{rr} S_{LS} & =\frac{(X^{T}R{)}^{2}}{{\hat{Var}}_{0}(X^{T}R|X,W)}\\ & =\frac{(X^{T}R{)}^{2}}{\left[X^{T}\left({\hat{\Sigma }}_{0}^{-1}-{\hat{\Sigma }}_{0}^{-1}W(W^{T}{\hat{\Sigma }}_{0}^{-1}W{)}^{-1}W^{T}{\hat{\Sigma }}_{0}^{-1}\right)X\right]}. \end{array}$$

The test statistic of the retrospective quasi-likelihood score test (denoted by $S_{QLS}$), also referred to as the MASTOR (Mixed-model Association Score Test On Related individuals) statistic [17], takes the form of

(6) $$\begin{array}{rr} S_{QLS} & =\frac{(R^{T}X{)}^{2}}{{\hat{Var}}_{0}\left(R^{T}X|Y,W\right)}\\ & =\frac{(R^{T}X{)}^{2}}{\left(R^{T}\Phi R\right){\hat{\sigma }}_{X}^{2}}. \end{array}$$

When Hardy–Weinberg equilibrium (HWE) is assumed at the SNP variant, a simple estimator of $\sigma_{X}^{2}$ can be obtained by ${\hat{\sigma }}_{X}^{2}=2\hat{p}(1-\hat{p})$, where $\hat{p}=\frac{1}{2}(1^{T}\Phi^{-1}1{)}^{-1}1^{T}\Phi^{-1}X$ is the best linear unbiased estimator (BLUE) [18] of the allele frequency p of X. Under the null, both $S_{LS}$ and $S_{QLS}$ follow a $\chi_{1}^{2}$ distribution.

### 2.2. Allele Frequency Estimates as Important Features for Association Testing

Allele frequencies play a fundamental role in population genetics and disease genetics studies. Accurate and reliable estimation of allele frequencies is essential in a wide range of applications, including population history inference [19,20], linkage analysis [21,22,23], association mapping [24,25], and admixture mapping [26,27]. At its core, GWAS seeks to answer a central question: whether allele frequencies at a given locus differ systematically across individuals with varying trait values or disease status. In genetic association testing involving samples with related individuals, allele frequency estimates provide critical information about the distribution of genotype data and play a key role in scaling test statistics, as illustrated in Equations (2) and (5) under the retrospective model. Consequently, within a machine learning framework for genetic association testing, allele frequency estimates can serve as informative features for identifying associated SNP variants.

Traditionally, in studies of unrelated individuals, allele frequencies can be estimated through simple naive counting. However, as more genetic studies include related individuals, naive counting estimators become disadvantageous or inappropriate, as they either restrict analysis to subsets of unrelated individuals (e.g., founders or singletons) or rely on misspecified models that assume independence among all sampled individuals. [28]. To address these limitations, several alternative approaches have been developed, including maximum likelihood estimation (MLE) [28,29,30] and generalized estimating equations (GEE) [31]. McPeek et al. further proposed the best linear unbiased estimator (BLUE) [18], which achieves computational efficiency for large and complex pedigrees while maintaining performance comparable to that of MLE.

In a pedigree-based genetic study, depending on whether parental information is available in the pedigree, the sampled individuals can be categorized into two groups: the founders (with parents not in the pedigree) and descendants (with parents in the pedigree). In what follows, we briefly introduce allele frequency estimators based on different sets of individuals in a pedigree-based study and explore some of their properties in Theorem 1.

**Theorem** **1.** *In a pedigree-based genetic study involving a group of n outbred individuals with a kinship matrix* Φ*, let *X  *be the genotype data collected for an SNP variant and let* ${\hat{p}}_{a},{\hat{p}}_{f},{\hat{p}}_{d}$  *denote estimators of allele frequency p, obtained by naive counting the number of alleles of type 1 in the set of all individuals, founders, and descendants, respectively. That is,*

(7) $${\hat{p}}_{a}=\frac{\sum_{i=1}^{n}X_{i}}{2n},{\hat{p}}_{f}=\frac{\sum_{i=1}^{n_{f}}X_{i}^{\left(f\right)}}{2n_{f}},{\hat{p}}_{d}=\frac{\sum_{i=1}^{n_{d}}X_{i}^{\left(d\right)}}{2n_{d}},$$

*where *$X_{i},X_{i}^{\left(f\right)},X_{i}^{\left(d\right)}$* denote the genotype of individual* i* in the set of all* n* individuals, the* $n_{f}$* founders, and the* $n_{d}$* descendants, respectively. Then, these estimators satisfy the following properties:*

(i) ${\hat{p}}_{a},{\hat{p}}_{f},{\hat{p}}_{d}$ are linear and unbiased estimators, and ${\hat{p}}_{a}=\frac{n_{f}}{n}{\hat{p}}_{f}+\frac{n_{d}}{n}{\hat{p}}_{d}$. Moreover, $var({\hat{p}}_{f})\leq var({\hat{p}}_{a})\leq var({\hat{p}}_{d})$; hence, the relative efficiencies $e({\hat{p}}_{a},{\hat{p}}_{f})\leq 1$ and $e({\hat{p}}_{a},{\hat{p}}_{d})\geq 1$

(ii) $cov({\hat{p}}_{f},{\hat{p}}_{d})=\frac{\sigma_{X}^{2}}{4n_{f}},\;cov({\hat{p}}_{a},{\hat{p}}_{f})=\frac{\sigma_{X}^{2}}{4n_{f}},\;cov({\hat{p}}_{a},{\hat{p}}_{d})=\frac{\sigma_{X}^{2}}{4n_{d}}\left(\frac{1^{T}\Phi 1}{n}-1\right)$, where $\sigma_{X}^{2}$ is the marginal variance of ***X*** and $\sigma_{X}^{2}=2p(1-p)$ under HWE. Hence, $cov({\hat{p}}_{a},{\hat{p}}_{d})$ is larger than $cov({\hat{p}}_{f},{\hat{p}}_{d})$ and $cov({\hat{p}}_{a},{\hat{p}}_{f})$

(iii) Among all linear unbiased estimators with form $(1-w){\hat{p}}_{f}+w{\hat{p}}_{d}$, ${\hat{p}}_{f}$ has the smallest variance.

The proofs of these properties are provided in Appendix A.

In the following deep-learning-based association testing methods, in particular DNN-LS-AF, DNN-QLS-AF, and DNN-ENS-AF (as defined in Section 2.3.1 and Section 2.3.2), we will use allele frequency estimators ${\hat{p}}_{f}$ and ${\hat{p}}_{d}$ as meaningful features of deep learning to predict test statistics or *p*-values and compare the results with those by training directly from the genotype data X.

### 2.3. Deep-Learning-Based Association Testing in Samples with Related Individuals

Artificial neural networks, particularly deep neural networks (DNNs), have become transformative tools in artificial intelligence and have been successfully applied across a wide range of domains, including computer vision, robotics, finance, and health care. By learning hierarchical representations through multiple layers of interconnected artificial neurons, DNNs are able to capture salient features and model complex patterns in data, enabling advanced predictive tasks, such as classification, regression, and representation learning [32,33,34]. With advances in modern computing technologies, deep learning paradigms have increasingly been introduced into GWAS, where they have shown promise in identifying and predicting disease-associated genetic variants [35].

Broadly speaking, deep learning approaches to genetic association testing can be categorized into two paradigms: model selection [36,37] and function approximation [38]. The model selection paradigm adopts a regression framework to characterize potentially nonlinear relationships between phenotypic traits and genotypic predictors, typically in an overparameterized and heuristic manner. Under this approach, DNNs are trained under both the null hypothesis (no association) and the alternative hypothesis (presence of association) using GWAS data. The resulting reduced and full models are then compared, and the model with superior goodness-of-fit (GoF) is selected based on predictive criteria, such as mean squared error (MSE), likelihood, or information criteria (e.g., AIC or BIC).

The function approximation paradigm is motivated by the universal approximation theorem [39,40], which guarantees that a sufficiently large or deep neural network can approximate any continuous function to an arbitrary degree of accuracy. Guided by this principle, DNNs can be trained to approximate association measures, such as test statistics or *p*-values for single variants or polygenic risk scores (PRS) for multiple variants across the genome. In addition to differences in network inputs and outputs, the function approximation paradigm also differs fundamentally from model selection in the nature of the training observations: model selection treats sampled individuals as observations, whereas function approximation treats genetic variants as observations.

In this work, we focus on the function approximation paradigm and propose several deep-learning-based association tests tailored to GWAS involving samples with related individuals. We refer to these methods as DNN-emulated association tests, or, equivalently, DNN-based emulators.

#### 2.3.1. DNN-Based Emulation of Association Testing

**Figure 1:**
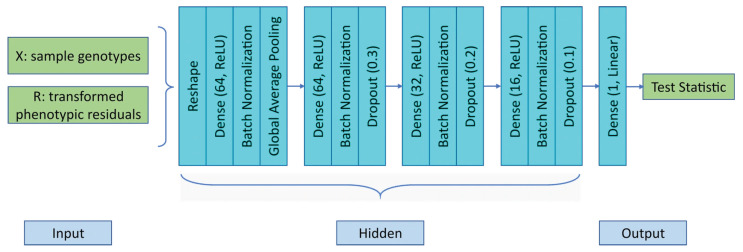
Network architecture of DNN-LS and DNN-QLS for simulated data.

The network architecture of our proposed DNN-based emulators, DNN-LS and DNN-QLS, is shown in Figure 1. This is a fully connected deep neural network with four hidden layers. The input includes genotype data X and the transformed phenotypic residual R of n (n=100 in our simulation) sampled individuals. We note that according to Equations (4) and (5), X and R play the role of sufficient statistics for conducting both the prospective LS and the retrospective QLS test. During the training procedure of these two original emulators, the input data are reshaped into an n×2 matrix. All four hidden layers are equipped with batch normalization to make training faster and more stable. Random dropout is adopted to prevent overfitting during training. The output is the test statistic from either Equation (4) or Equation (5). To avoid variance shrinkage in predicting the test statistic, the MSE loss function is supplemented with a variance preserving term, which accounts for possible variance change after prediction. It is noteworthy that in addition to the test statistic, the resulting *p*-value may also be treated as the output, though this will increase the difficulty of approximation. Moreover, when the true association signal, i.e., the binary label indicating whether a variant is associated with the trait, is available, this label information or the probability of a variant being associated with the trait can be used as the output, as well. Table 1 shows the hyperparameter settings of the DNN-based emulators.

In addition, we considered four other variations of DNN-based emulators, namely, DNN-LS-RAW, DNN-QLS-RAW, DNN-LS-AF, and DNN-QLS-AF. DNN-LS-RAW and DNN-QLS-RAW treated the raw data of X, two covariates $W_{1},W_{2}$, and trait Y as the input, while ignoring the relatedness information among the sampled individuals. Theoretically, the kinship matrix Φ can also be incorporated into the input; however, this will add additional n2 features to the input. With the number of input features increased so drastically, the DNN architecture will be too complicated, and the convergence of training cannot be guaranteed. In their current setting, DNN-LS-RAW and DNN-QLS-RAW added an additional hidden layer of 128 neurons (with batch normalization and a dropout rate of 0.4) between the first and second hidden layers in Figure 1. DNN-LS-AF and DNN-QLS-AF, on the other hand, simplified the input by using $X^{T}R$ together with two estimated allele frequencies ${\hat{p}}_{f},{\hat{p}}_{d}$ (as introduced in Section 2.2). A single hidden layer with eight neurons was used to handle these three input features.

#### 2.3.2. DNN-Based Ensemble Learning

**Figure 2:**
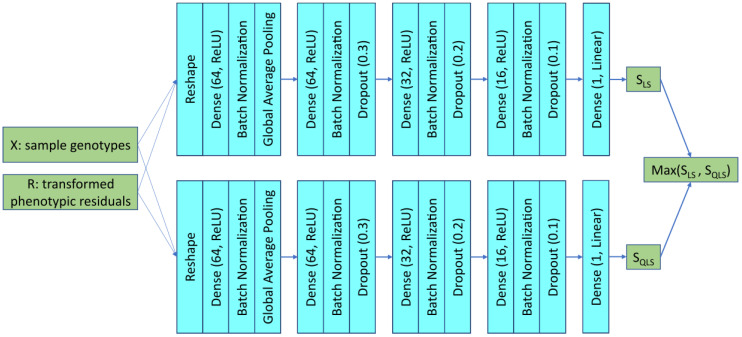
Network architecture of DNN-ENS for simulated data.

The DNN-based emulation can be extended by ensemble learning. The key idea is to combine multiple predictive models, e.g., DNN-LS and DNN-QLS, to improve predictive performance. Here, we adopted the bagging (Bootstrap Aggregating) strategy. By aggregating predictions from the two DNN-based emulators, the ensemble emulator, called DNN-ENS, is expected to achieve better accuracy, robustness, and generalization. Figure 2 shows the network architecture of DNN-ENS, where the major change lies in the output—for ensemble learning, the output is the maximum of the LS Equation (4) and QLS statistics Equation (5).

Ensemble learning was also applied to DNN-LS-AF and DNN-QLS-AF, resulting in an alternative ensemble emulator, DNN-ENS-AF.

## 3. Results

### 3.1. Simulation

We perform simulations to evaluate the proposed deep-learning-based association tests in three aspects, as listed in the following subsections.

#### 3.1.1. Emulating Prospective and Retrospective Association Tests: Using Transformed Phenotypic Residual vs. Using Raw Data

Our simulated data were generated from the following linear mixed model:

(8) $$\begin{array}{r} y=W\beta +x\alpha +\epsilon \end{array}$$

where y is a length-*n* vector of the quantitative trait in n sampled individuals, W is a n×q covariate matrix (intercept included) with fixed effects β, x contains genotypes of a given SNP variant, which is possibly associated with y through a scalar coefficient α, and ϵ is the error term following a N(0,Σ) distribution with $\Sigma =\sigma_{e}^{2}I+\sigma_{a}^{2}\Phi$.

**Figure 3:**
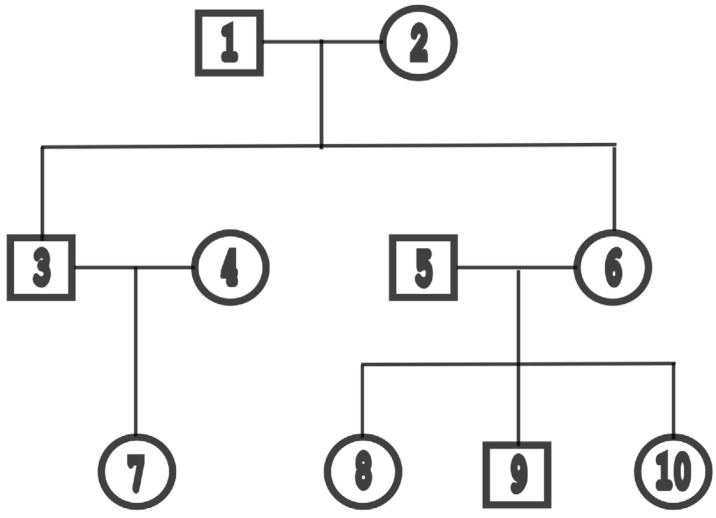
Basic family structure of 10 individuals coming from three generations, used in simulations for generating data in samples with related individuals.

In the simulation, we sampled n=100 individuals from 10 three-generation families, each containing 10 individuals who are related, as in Figure 3. We let q=3, so that W contains two nongenetic covariates, age $W_{1}$ and gender $W_{2}$, which were drawn from the uniform(18, 80) and Bernoulli(0.7) distributions, respectively. The regression coefficients were set as $\beta ={\left(1,0.5,0.8\right)}^{T}$, and the variance components were set as $\sigma_{e}^{2}=1.5,\sigma_{a}^{2}=2.5$. Genotype data x were generated by gene-dropping along the three generations in each family. The emulation of the LS and QLS tests is based on a multi-layer perceptron (MLP) neural network with four fully connected hidden layers (see Figure 1 for network architecture and Table 1 for hyperparameter settings).

**Figure 4:**
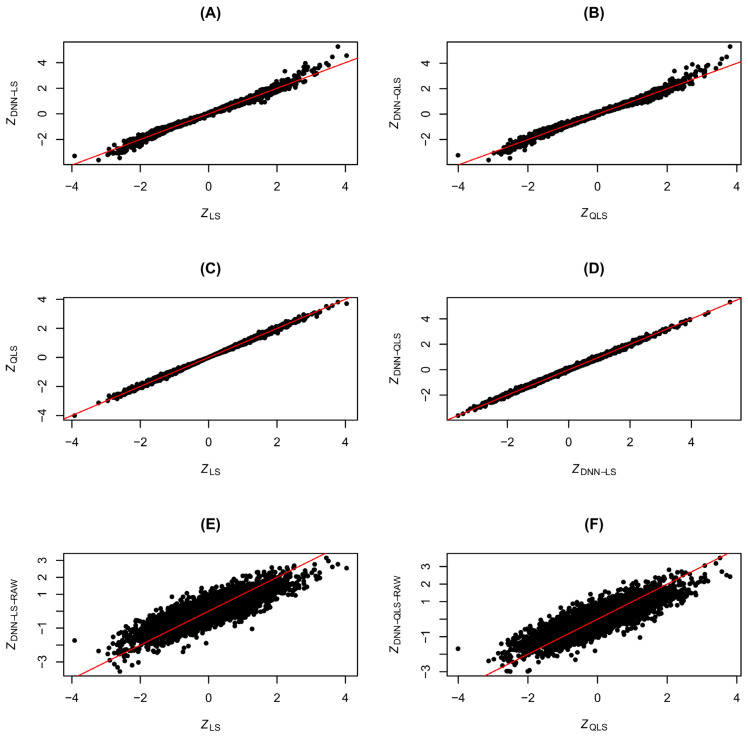
Consistency check for association tests under *H*0: (**A**) *Z_LS_* vs. *Z_DNN_*_-_*_LS_*, (**B**) *Z_QLS_* vs. *Z_DNN_*_-_*_QLS_*, (**C**) *Z_LS_* vs. *Z_QLS_*, (**D**) *Z_DNN_*_-_*_LS_* vs. *Z_DNN_*_-_*_QLS_*, (**E**) *Z_LS_* vs. *Z_DNN_*_-_*_LS_*_-_*_RAW_*, (**F**) *Z_QLS_* vs. *Z_DNN_*_-_*_QLS_*_-_*_RAW_*. *Z_LS_*: Z statistic of likelihood score test; *Z_QLS_*: Z statistic of quasi-likelihood score test; *Z_DNN_*_-_*_LS_*: Z statistic of DNN-emulated likelihood score test; *Z_DNN_*_-_*_QLS_*: Z statistic of DNN-emulated quasi-likelihood score test; *Z_DNN_*_-_*_LS_*_-_*_RAW_*: Z statistic of DNN-emulated likelihood score test using raw data; *Z_DNN_*_-_*_QLS_*_-_*_RAW_*: Z statistic of DNN-emulated quasi-likelihood score test using raw data. The black dots represent pairs of test statistic values, and the diagonal red lines represent *y* = *x*.

**Figure 5:**
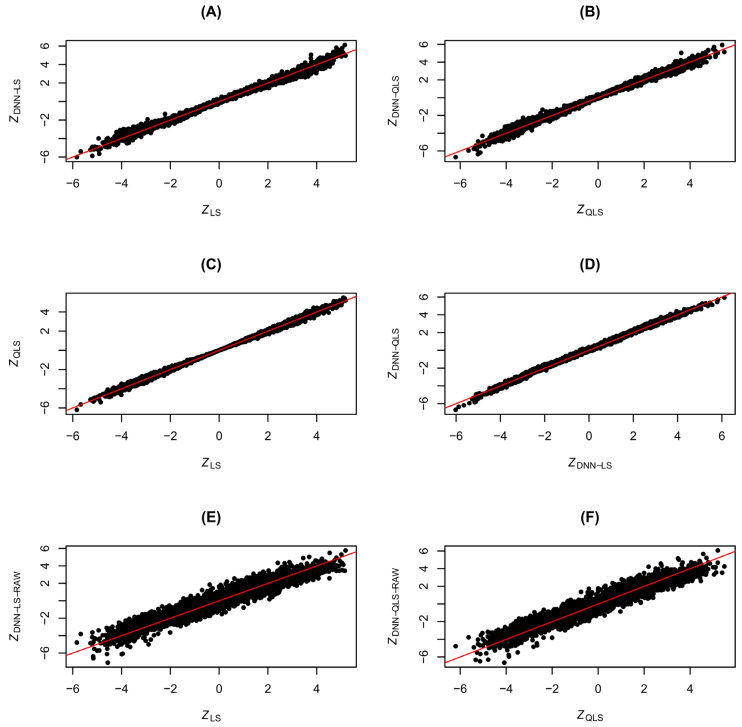
Consistency check for the four association tests under *Ha*: (**A**) *Z_LS_* vs. *Z_DNN_*_-_*_LS_*, (**B**) *Z_QLS_* vs. *Z_DNN_*_-_*_QLS_*, (**C**) *Z_LS_* vs. *Z_QLS_*, (**D**) *Z_DNN_*_-_*_LS_* vs. *Z_DNN_*_-_*_QLS_*, (**E**) *Z_LS_* vs. *Z_DNN_*_-_*_LS_*_-_*_RAW_*, (**F**) *Z_QLS_* vs. *Z_DNN_*_-_*_QLS_*_-_*_RAW_*. *Z_LS_*: Z statistic of likelihood score test; *Z_QLS_*: Z statistic of quasi-likelihood score test; *Z_DNN_*_-_*_LS_*: Z statistic of DNN-emulated likelihood score test; *Z_DNN_*_-_*_QLS_*: Z statistic of DNN-emulated quasi-likelihood score test; *Z_DNN_*_-_*_LS_*_-_*_RAW_*: Z statistic of DNN-emulated likelihood score test using raw data; *Z_DNN_*_-_*_QLS_*_-_*_RAW_*: Z statistic of DNN-emulated quasi-likelihood score test using raw data. The black dots represent pairs of test statistic values, and the diagonal red lines represent y = x.

We first checked the consistency between LS and DNN-LS and between QLS and DNN-QLS. For comparison purposes, DNN-LS-RAW and DNN-QLS-RAW were also included in the consistency check. In the simulation, we generated genotype data for 5000 SNP variants, with the minor allele frequency (MAF) p sampled from uniform (0.1, 0.5). The genotype association effect α in model (6) was determined in two conditions: under $H_{0},\;\alpha \;=\;0$, and, under $H_{a},\;\alpha$ was drawn from a uniform distribution supported on [−1.25,−0.25]⋃[0.25,1.25]. Using data generated from (6), we tested the genetic association for each SNP variant one by one and made a straight comparison for the test statistics from different tests: LS, DNN-LS, DNN-LS-RAW, QLS, DNN-QLS, and DNN-QLS-RAW. The consistency check under $H_{0}$ and under $H_{a}$ is visualized in Figure 4 and Figure 5, respectively. For ease of approximation and comparison, the test statistics in the demonstration were chosen to be of a Z-statistic form and denoted as $Z_{LS}$, $Z_{DNN-LS}$, $Z_{DNN-LS-RAW}$, $Z_{QLS}$, $Z_{DNN-QLS}$, and $Z_{DNN-QLS-RAW}$ (e.g., $Z_{LS}=sign(X^{T}R)\cdot \sqrt{S_{LS}}$). For comparison between the $\chi^{2}$ statistics and between the resulting *p*-values, please check Supplementary Material. From Figure 4A,B and Figure 5A,B it can be seen that the consistency between $Z_{LS}$ and $Z_{DNN-LS}$ and between $Z_{QLS}$ and $Z_{DNN-QLS}$ is pretty high, indicating that, in general, the traditional LS and QLS tests can be emulated effectively by DNN. In contrast, Figure 4E,F and Figure 5E,F tell us that the performance of DNN-based emulation could be poor if we chose DNN-LS-RAW or DNN-QLS-RAW, because these DNN models were misspecified (relatedness was discarded) and had too many learnable weights (for a total of 4n features). For completeness of the comparison, we also show the scatter plots of $Z_{LS}$ VS. $Z_{QLS}$ in Figure 4C and Figure 5C, and show the scatter plots of $Z_{DNN-LS}$ VS. $Z_{DNN-QLS}$ in Figure 4D and Figure 5D. The results in Figure 4C and Figure 5C confirmed the consistency between LS and QLS, which has been found in the literature [17], and, consequently, it is not surprising to see the high consistency between DNN-LS and DNN-QLS in Figure 4D and Figure 5D. The QQ-plots of the test statistics $Z_{LS}$, $Z_{QLS}$, $Z_{DNN-LS}$, $Z_{DNN-QLS}$, $Z_{DNN-LS-RAW}$, and $Z_{DNN-QLS-RAW}$ under $H_{0}$ are included in Supplementary Material, showing that the null distributions of $S_{LS}$ and $S_{QLS}$ follow exactly $\chi_{1}^{2}$ (and those of $S_{DNN-LS}$, $S_{DNN-QLS}$, $S_{DNN-LS-RAW}$, and $S_{DNN-QLS-RAW}$ follow approximately $\chi_{1}^{2}$).

The approximation accuracy of these DNN emulators is summarized in Table 2 in terms of mean absolute error (MAE), root mean squared error (RMSE), R-squared, and correlation. These measures clearly show that DNN-LS and DNN-QLS outperform DNN-LS-RAW and DNN-QLS-RAW in approximating the Z-statistic (or the $\chi^{2}$-statistic and the *p*-value) functions of the association tests under both the null and the alternative hypotheses. Comparing the R-squared and correlation measures between the two columns in Table 2, we also see that the approximation under $H_{a}$ is generally better than under $H_{0}$. This shows that it is relatively easier for DNN to approximate the test statistics in the presence of salient association signals.

These simulations were then used to evaluate the six testing methods, LS, QLS, DNN-LS, DNN-QLS, DNN-LS-RAW, and DNN-QLS-RAW, in terms of type I errors and power. Under $H_{0}$, the genotype association effect α was set to 0, whereas under $H_{a}$ α was drawn from a uniform distribution supported on [−0.5(k+1),−0.5k]⋃[0.5k,0.5(k+1)] where integer k varies at different values 1, 1.5, 2, 2.5, corresponding to four levels of associations from weak to strong. Using data simulated for 5000 SNPs, we performed association testing using LS, QLS, and their DNN emulators and calculated the empirical type I error rates and power, as listed in Table 3. Note that for this simulation and those hereinafter, the entire simulated dataset was divided into training and test sets according to a ratio 4:1, and the calculation of type I errors and power is based on predictions on the test set. From Table 3, we first observe that LS, QLS, DNN-LS, and DNN-QLS attain correctly controlled type I errors, whereas DNN-LS-RAW and DNN-QLS-RAW are too conservative in calculating the type I error due to model misspecification caused by the ignored relatedness information. In light of this observation, together with the approximation accuracy shown in Figure 4E,F and Figure 5E,F and Table 2, we conclude that DNN-LS-RAW and DNN-QLS-RAW are not suitable models to learn the association rule as they are both over-parameterized and misspecified. We therefore excluded DNN-LS-RAW and DNN-QLS-RAW from our follow-up analyses. It can also be seen that for most of the settings on the true association effect, DNN-LS and DNN-QLS achieve power comparable to or higher than the original LS and QLS tests. This shows that these two DNN-emulated tests are good at detecting association signals from learnable patterns in GWAS data.

#### 3.1.2. Incorporating Allele Frequency Estimates into DNN-Emulated Association Tests

As described in Section 2.3.1, DNN-LS-AF and DNN-QLS-AF used more simplified input features than DNN-LS and DNN-QLS. This was done by replacing genotype data X and the transformed phenotypic residual R with their inner product $X^{T}R$ and two estimated allele frequencies ${\hat{p}}_{f},{\hat{p}}_{d}$, which reduced the dimensionality of input features from 2n to 3. The performance of DNN-LS-AF and DNN-QLS-AF is shown in Table 4. From this table, we see that DNN-LS-AF and DNN-QLS-AF demonstrate power comparable to DNN-LS and DNN-QLS while still keeping the type I error well-controlled. This shows that using meaningful features (with invariant properties) in deep learning generally helps detect genetic associations. This is further confirmed by the approximation accuracy of DNN-LS-AF and DNN-QLS-AF in the Supplementary Material. The last column of Table 4 reports the total training time under five settings k={0, 1, 1.5, 2, 2.5} on a laptop with i7 2.5 GHz CPU and 16 G RAM. It is not surprising that DNN-LS-AF and DNN-QLS-AF exhibit reduced training costs, given their substantially simplified network architectures.

#### 3.1.3. Ensemble Learning from Prospective and Retrospective Association Tests

The evaluation of two ensemble emulators, DNN-ENS and DNN-ENS-AF, is provided in Table 5. For comparison, the type I error and the power of the LS and QLS tests are also included in this table. In general, we see that ensemble learning turns out to be relatively more powerful than LS or QLS, while correctly controlling the type I error. Similar to the results shown in Table 4, using estimated allele frequencies as input features within an ensemble learning framework achieves comparable power while substantially simplifying the network architecture.

### 3.2. Real Data Example

The Framingham Heart Study (FHS) [41] is a long-term GWAS initiated in 1948 with the goal of identifying risk factors for cardiovascular disease (CVD). The FHS comprises three cohorts—the Original, Offspring, and Third Generation cohorts—for which genotypic and phenotypic data have been collected from 1538 multigenerational pedigrees. Our analysis focuses on genome-wide associations between common SNPs and systolic blood pressure (SBP). For each of the 14,173 individuals, we computed a quantitative trait by averaging multiple SBP measurements across examinations. To account for the effects of antihypertensive medication, SBP measurements obtained while on treatment were adjusted by adding a constant of 10 mmHg [42,43], and the adjusted values were subsequently log-transformed. Nine clinical variables were included as covariates: sex, age, age^2^, log-transformed body mass index (BMI; MIM: 606641), log-transformed current smoking (defined as the number of cigarettes smoked per day), smoking history (coded as 1 for current or former smokers and 0 for never smokers), log-transformed blood glucose, and two cohort indicator variables. Genotype data were obtained for 9240 individuals using the Affymetrix 500 K array. To ensure reliable association results, individuals were excluded during quality control if they (1) had an empirical self-kinship coefficient ≥ 0.525 or (2) had genotype completeness ≤ 96%, where completeness was defined as the proportion of successfully genotyped markers per individual. We further excluded low-quality or inappropriate SNPs based on the following criteria: (1) call rate < 96%, (2) Mendelian error rate > 2%, or (3) minor allele frequency (MAF) < 1%, corresponding to rare variants.

We first performed a whole-genome analysis of genetic associations on average SBP measurements from the FHS using the classical LS and QLS tests. This analysis was based on a total of 8087 sampled individuals who met the quality-control criteria and were both genotyped and phenotyped. Among these individuals, 7854 were from 872 multigeneration pedigrees and 233 were apparently unrelated. Statistical tests found 2547 SNPs with *p*-values < 0.05 reported by both LS and QLS. We then trained a DNN-ENS-AF model using the LS and QLS statistics of these SNPs that are significant at the single-variant level, and obtained their predicted *p*-values by ensemble emulation. Table 6 lists the SNPs for which both QLS and DNN-ENS-AF gave *p*-values lower than a strict significance threshold of $2.5\times 10^{-5}$. Note that the *p*-values obtained by the LS test were not incorporated here as they appeared to be generally larger than the *p*-values obtained by the QLS test. We see that for all these 12 SNPs in Table 6, DNN-ENS-AF shows smaller *p*-values than QLS. This confirms our conclusion from the simulation study that DNN-emulated tests are good at detecting association signals from learnable patterns in GWAS data. By the way, we note that this real data analysis is different from that of Wu and McPeek, 2018 [44], in two major aspects: (1) the former used data from all three cohorts whereas the latter excluded data from cohort 1, and (2) the former considered average SBP measurements whereas the latter used longitudinal SBP measurements. Nevertheless, the detected association signals from our analysis and from Wu and McPeek, 2018 [44] still show a high consistency. This can be seen from the bold-faced genes *PIK3CG* and *C10orf107* in Table 6, which was identified in the literature as associated with pulse pressure and/or the difference between SBP and diastolic blood pressure (DBP) in meta-analyses of individuals of European ancestry [45,46,47]. In addition, the scatter plots of Z-statistics of the LS, QLS, and DNN-ENS-AF tests for the 2547 SNPs with *p*-value < 0.05 is shown in Figure S6 of Supplementary Material. These figures confirm that overall, DNN-ENS-AF performs similarly to QLS (LS is clearly underpowered), while training on SNPs that are significant at the single-variant level helps produce magnified values for test statistics falling in the critical region (i.e., those on the tail with extreme values), as shown in Figure S6B.

## 4. Discussion

Machine learning ML and deep learning DL extend GWAS beyond marginal association testing by enabling joint modeling, capturing nonlinear effects, and facilitating integrative analyses. Despite their increasing applications in GWAS, the fundamental question of how ML and DL can directly aid or improve genetic association testing remains underexplored, particularly for GWAS involving high-dimensional data with complex correlations arising from population stratification or cryptic relatedness. In this study, we propose a DNN-aided approach for testing genetic associations in samples with related individuals. The core idea is to train DNN models to approximate the relationship between genotype and phenotype using pedigree-based GWAS data with known or tested association effects. Depending on the input features, several variations of DNN-emulated tests are considered: DNN-LS and DNN-QLS use genotype data along with transformed phenotypic residuals; DNN-LS-RAW and DNN-QLS-RAW use raw genotype and phenotype data while ignoring relatedness; DNN-LS-AF and DNN-QLS-AF incorporate allele frequency estimators to reduce dimensionality; and DNN-ENS and DNN-ENS-AF further aggregate predictions by combining multiple emulators. Through simulations, we demonstrate that DNN-emulated tests effectively approximate traditional test statistics and are powerful in identifying associated variants, with the exception of DNN-LS-RAW and DNN-QLS-RAW, which suffer from overparameterization and model misspecification. Moreover, replacing raw data with meaningful features or summary statistics improves computational efficiency, and ensemble learning enhances prediction reliability. When applied to GWAS data from the FHS, the proposed approach identifies SNP variants in or near genes previously reported to be associated with blood pressure, including *PIK3CG* and *C10orf107*, achieving relatively lower *p*-values.

It is often argued that the function-approximation approach for association testing may appear logically contradictory, as it seems counterintuitive that an emulator could outperform the original statistical model. However, with the complex data generated by modern GWAS technologies, classical statistical models often encounter challenges arising from sporadic missingness or latent structures and correlations, which can lead to inflated testing errors and reduced power. Deep learning excels at extracting meaningful patterns from irregular, noisy, or high-dimensional data by selecting, tuning, and optimizing the weights of large, multi-layered neural networks, thereby addressing difficulties that classical association tests struggle with. Nevertheless, it is important to recognize that due to the lack of statistical rigor, ML and DL models should be best viewed as complementary tools rather than replacements for classical association tests.

We note that in our real data analysis, the DNN model was trained using SNPs that were significant at the single-variant level. This ensures that the association signal is reinforced during training. In practice, it is also possible to train separate models for SNPs with significant versus non-significant *p*-values at the single-variant level. For example, a mixture-of-experts (MOE) model [48] can be used to learn patterns separately for SNPs with and without genetic associations, thereby improving the ability to distinguish true signals from noise.

DNN-aided association testing can also be used to directly predict whether a variant is associated with a trait. Because in real GWAS the true phenotype–genotype relationship is typically unknown, a common strategy is transfer learning, in which predictions are made by leveraging pre-trained models derived from GWAS summary statistics, such as those available in the GWAS Catalog [1] or GWAS Atlas [49]. This approach accelerates training, enhances predictive performance, and requires less data to achieve high accuracy.

Finally, the proposed approach is highly flexible and can be extended in multiple ways. For example, by including the LD score (e.g., the average r2 between the target variant and all other variants located within a neighborhood) of each SNP as an input feature, the DNN emulators may gain additional power in association testing by accounting for the contribution of causal loci involved in epistasis. Other potential extensions include modeling interaction effects for multi-variant association testing and accommodating multiple or heterogeneous traits in pleiotropy analyses.

## Figures and Tables

**Table 1:** Summary of hyperparameter settings for DNN-LS and DNN-QLS.

Hyperparameters	Values
Epochs	200
Batch size	16
Initial learning rate	0.001
Dropout rate	0.1 ~ 0.3
L1 regularization	0.01
Number of hidden neurons	(64, 64, 32, 16)
Optimizer	Adam
Activation function	ReLU except the output layer (linear)

**Table 2:** Summary of approximation accuracy of DNN-LS, DNN-QLS, DNN-LS-RAW, and DNN- QLS-RAW.

	$\mathrm{Under}\;H_{0}$				$\mathrm{Under}\;H_{a}$			
	DNN- LS	DNN- QLS	DNN- LS- RAW	DNN- QLS- RAW	DNN- LS	DNN- QLS	DNN- LS- RAW	DNN- QLS- RAW
MAE	0.0747	0.0785	0.3982	0.3940	0.1590	0.1556	0.4426	0.4461
RMSE	0.1121	0.1180	0.5048	0.4994	0.2174	0.2090	0.5607	0.5668
R^2^	0.9878	0.9861	0.7521	0.7515	0.9913	0.9919	0.9423	0.9402
Correlation	0.9939	0.9931	0.8683	0.8676	0.9960	0.9963	0.9712	0.9700

**Table 3:** Evaluation of different association tests.

Tests	Type I Error		Power *		
		*k* = 1	*k* = 1.5	*k* = 2	*k* = 2.5
LS	0.051	0.537	0.750	0.883	0.962
QLS	0.046	0.526	0.739	0.876	0.954
DNN-LS	0.047	0.583	0.733	0.920	0.961
DNN-QLS	0.048	0.558	0.736	0.893	0.963
DNN-LS-RAW	0.019	0.557	0.792	0.928	0.986
DNN-QLS-RAW	0.007	0.541	0.757	0.939	0.982

* Under *H_a_*, the association effect was drawn from a uniform distribution supported on [−0.5(k+1),−0.5k]⋃[0.5k,0.5(k+1)]. Thus, *k* controls the effect size and *k* = 1, 1.5, 2, 2.5 correspond to four different levels of associations from weak to strong.

**Table 4:** Evaluation of variations of DNN-emulated association tests.

Tests	Type I Error	Power				Training Time *
		*k* = 1	*k* = 1.5	*k* = 2	*k* = 2.5	(s)
DNN-LS	0.047	0.583	0.733	0.920	0.961	492.52
DNN-QLS	0.048	0.558	0.736	0.893	0.963	491.07
DNN-LS-AF	0.050	0.577	0.739	0.889	0.951	382.93
DNN-QLS-AF	0.048	0.549	0.718	0.886	0.965	353.68

* Total training time for *k* takes values in {0, 1, 1.5, 2, 2.5}, based on 4000 training SNPs.

**Table 5:** Evaluation of DNN-emulated association tests by ensemble learning.

Tests	Type I Error	Power			
		*k* = 1	*k* = 1.5	*k* = 2	*k* = 2.5
LS	0.051	0.537	0.750	0.883	0.962
QLS	0.046	0.526	0.739	0.876	0.954
DNN-ENS	0.051	0.591	0.752	0.905	0.951
DNN-ENS-AF	0.055	0.572	0.744	0.900	0.966

**Table 6:** Strongest association signals in the FHS SBP data.

Chromosomal Region	Nearby Genes	SNP ID	Position	*p*-Values *		
				LS	QLS	DNN-ENS-AF
2q12	*LINC01593*	rs3895955	108,053,441	1.86 × 10^−3^	8.60 × 10^−6^	4.55 × 10^−7^
3p24	*RBMS3*	rs9877765 rs4630899	29,665,389	4.47 × 10^−3^	1.55 × 10^−5^	3.33 × 10^−7^
			29,674,590	3.69 × 10^−3^	1.02 × 10^−5^	1.68 × 10^−7^
3p13	*LINC00877*	rs11710880	72,214,965	3.09 × 10^−3^	2.14 × 10^−5^	7.93 × 10^−6^
5p15	*MIR4277*	rs16883212	1,742,029	2.96 × 10^−3^	2.15 × 10^−5^	1.03 × 10^−6^
7q22	***PIK3CG***	rs17398575	106,196,688	1.08 × 10^−3^	3.69 × 10^−6^	7.47 × 10^−7^
		rs12705390	106,198,013	6.61 × 10^−4^	1.27 × 10^−6^	2.94 × 10^−7^
	*FLJ36031*	rs11760498	106,206,208	3.58 × 10^−3^	2.40 × 10^−5^	2.38 × 10^−5^
10q21	***C10orf107***	rs12246717	63,129,189	2.46 × 10^−3^	1.53 × 10^−5^	3.59 × 10^−6^
12q21	*ATP2B1*	rs17249754	88,584,717	7.30 × 10^−4^	2.78 × 10^−6^	9.70 × 10^−7^
14q31	*GALC*	rs450711	87,498,297	2.38 × 10^−3^	1.46 × 10^−5^	2.83 × 10^−7^
21q22	*SLC37A1*	rs449888	42,877,061	2.20 × 10^−3^	1.49 × 10^−5^	4.15 × 10^−7^

* SBP-associated SNPs with both *p*-values from QLS and DNN-ENS-AF *<* 2.5 × 10^−5^ are reported on the basis of the FHS data. Bold-faced genes have been previously identified in the literature as associated with SBP.

## Data Availability

The Framingham Heart Study (FHS) is conducted and supported by the National Heart, Lung, and Blood Institute (NHLBI) in collaboration with Boston University (contract nos. N01-HC-25195 and HHSN268201500001I). The Framingham SHARe data used for the analyses described in this manuscript were obtained through dbGaP: phs000007/HMB-IRB-MDS and phs000007/HMB-IRB-NPU-MDS. This manuscript was not prepared in collaboration with investigators of the FHS and does not necessarily reflect the opinions or views of the FHS, Boston University, or the NHLBI. The original contributions presented in this study are included in the article and Supplementary Material. Further inquiries can be directed to the corresponding author(s).

## Supplementary Materials

The following supporting information can be downloaded at https://www.mdpi.com/article/10.3390/cimb48030273/s1. The source code of the proposed method is publicly available at https://github.com/XiaoweiWu-VT/DNN-GWAS (accessed on 26 February 2026).

## Abbreviations

The following abbreviations are used in this manuscript:

GWAS	Genome-wide association study
SNP	Single nucleotide polymorphism
LMM	Linear mixed model
VCM	Varying coefficient model
GEE	Generalized estimating equation
FDA	Functional data analysis
SKAT	Sequence kernel association tests
ML	Machine learning
DL	Deep learning
LS	Likelihood score
QLS	Quasi-likelihood score
MASTOR	Mixed-model association score test on related individuals
HWE	Hardy-Weinberg equilibrium
BLUE	Best linear unbiased estimator
MLE	Maximum likelihood estimation
DNN	Deep neural network
GoF	Goodness-of-fit
MSE	Mean squared error
AIC	Akaike information criterion
BIC	Bayesian information criterion
PRS	Polygenic risk score
MLP	Multi-layer perceptron
MAF	Minor allele frequency
MAE	Mean absolute error
RMSE	Root mean squared error
FHS	Framingham heart study
CVD	Cardiovascular disease
SBP	Systolic blood pressure
BMI	Body mass index
DBP	Diastolic blood pressure
MOE	Mixture-of-experts

## Appendix A. Lemma A1 and Proof of Theorem 1

**Lemma** **A1.** *For outbred individuals, the kinship matrix*  Φ *satisfies the following two inequalities:*

$$(1^{T}\Phi 1)(1^{T}\Phi^{-1}1)\geq n^{2},\;1^{T}\Phi 1+1^{T}\Phi^{-1}1\geq 2n.$$

**Proof.** Let $u=\Phi^{1/2}1$ and $v=\Phi^{-1/2}1$. First, by the Cauchy–Schwarz inequality $(u^{T}u)(v^{T}v)\;\geq \;{\left(u^{T}v\right)}^{2},\;(1^{T}\Phi 1)(1^{T}\Phi^{-1}1)\;\geq \;n^{2}$. Next, easy to see by linear algebra that $u^{T}u+\;v^{T}v\geq \;2u^{T}v$**.** Therefore, $1^{T}\Phi 1+1^{T}\Phi^{-1}1\geq 2n$. □

**Proof of Theorem** **1:** We note that ***X*** is a multivariate binomial random vector, and $E[X]=2p1,cov(X)=\sigma_{X}^{2}\Phi$.

(i) From the definitions of these allele frequency estimators, we see that

(A1)
$${\hat{p}}_{a}=\frac{n_{f}}{n}{\hat{p}}_{f}+\frac{n_{d}}{n}{\hat{p}}_{d}.$$

In vector form, these allele frequency estimators can be defined by

(A2) $${\hat{p}}_{a}=\frac{1}{2}(1^{T}1{)}^{-1}1^{T}X,\;{\hat{p}}_{f}=\frac{1}{2}(Z_{f}^{T}1{)}^{-1}Z_{f}^{T}X,\;{\hat{p}}_{d}=\frac{1}{2}(Z_{d}^{T}1{)}^{-1}Z_{d}^{T}X$$

where ***Z_f_*** is an length-*n* binary vector indicating the group of the individuals (i.e., “1”: founder; “0”: descendant) and ***Z_d_*** = **1** − ***Z_f_***. It is clear that all three estimators ${\hat{p}}_{a},{\hat{p}}_{f}$, and ${\hat{p}}_{d}$ are linear (defined as taking the form ***a^T^X***). The unbiasedness of these estimators is straightforward.

For outbred individuals, it can be seen that $Z_{f}=\Phi^{-1}1$, and therefore ${\hat{p}}_{f}=\frac{1}{2}(1^{T}\Phi^{-1}1{)}^{-1}1^{T}\Phi^{-1}X$. The variances of these estimators are consequently obtained:

(A3) $$\begin{array}{rr} var({\hat{p}}_{a}) & =\;\frac{\sigma_{X}^{2}}{4n^{2}}1^{T}\Phi 1,\\ var({\hat{p}}_{f}) & =\;\frac{1}{4}(Z_{f}^{T}1{)}^{-2}\sigma_{X}^{2}(Z_{f}^{T}\Phi Z_{f})=\frac{\sigma_{X}^{2}}{4n_{f}},\\ var({\hat{p}}_{d}) & =\;\frac{1}{4}(Z_{d}^{T}1{)}^{-2}\sigma_{X}^{2}(Z_{d}^{T}\Phi Z_{d})=\frac{\sigma_{X}^{2}}{4n_{d}^{2}}(1^{T}\Phi 1-n-n_{d}) \end{array}$$

where $\sigma_{X}^{2}$ is the variance of ***X***, and $\sigma_{X}^{2}=2p(1-p)$ under HWE.

To see their relative efficiencies, first, by the first inequality in Lemma A1, $var({\hat{p}}_{a})\geq var({\hat{p}}_{f})$. Next, since $1^{T}\Phi^{-1}1=n_{f}<2n$, multiplying a non-positive term $n^{2}-(1^{T}\Phi 1)(1^{T}\Phi^{-1}1)$ on both sides leads to $\left(1^{T}\Phi^{-1}1)[n^{2}-(1^{T}\Phi 1)(1^{T}\Phi^{-1}1)]\geq 2n[n^{2}-(1^{T}\Phi 1)(1^{T}\Phi^{-1}1)\right]$. Rearranging the terms, we obtain $n^{2}(1^{T}\Phi 1-2n+1^{T}\Phi^{-1}1)\geq (1^{T}\Phi 1)(n-1^{T}\Phi^{-1}1{)}^{2}$, which then simplifies to $\frac{1^{T}\Phi 1-n-n_{d}}{n_{d}^{2}}\geq \frac{1^{T}\Phi 1}{n^{2}}$. That is, $var({\hat{p}}_{d})\geq var({\hat{p}}_{a})$.

It follows directly that

$$e({\hat{p}}_{a},{\hat{p}}_{f})=\frac{n^{2}}{n_{f}(1^{T}\Phi 1)}\leq 1$$

and

$$e({\hat{p}}_{a},{\hat{p}}_{d})=\frac{n^{2}(1^{T}\Phi 1-n-n_{d})}{n_{d}^{2}(1^{T}\Phi 1)}\geq 1.$$

(ii) By Equation (A1),

(A4)
$$var({\hat{p}}_{a})=\frac{n_{f}^{2}}{n^{2}}var({\hat{p}}_{f})+\frac{n_{d}^{2}}{n^{2}}var({\hat{p}}_{d})+2\frac{n_{f}n_{d}}{n^{2}}cov({\hat{p}}_{f},{\hat{p}}_{d}).$$

Plugging in the variance expressions in (A3), we obtain $cov({\hat{p}}_{f},{\hat{p}}_{d})=\frac{\sigma_{X}^{2}}{4n_{f}}$. The other two covariances follow directly. By the Cauchy-Schwarz inequality, it can be seen that $cov({\hat{p}}_{a},{\hat{p}}_{d})$ is larger than the other two.

(iii) It follows directly from the variance and covariance expressions in (i) and (ii) that, among all linear unbiased estimators with form $(1-w){\hat{p}}_{f}+w{\hat{p}}_{d}$, ${\hat{p}}_{f}$ has the smallest variance (which happens when *w* = 0). This confirms the BLUE property [18] of ${\hat{p}}_{f}$. □
