# Supplementary material for: Deep-Neural-Network-Aided Genetic Association Testing in Samples with Related Individuals
Source: Curr Issues Mol Biol. 2026 Mar 4;48(3):273. doi: 10.3390/cimb48030273 (PMC13025615; doi:10.3390/cimb48030273)
Supplement: Supplementary file 1 [file cimb-48-00273-s001.zip › cimb-4162847-supplementary.pdf]

Supplementary Materials for "Deep neural network-aided genetic association testing in samples with related individuals"

- **Supplementary figures in simulation**

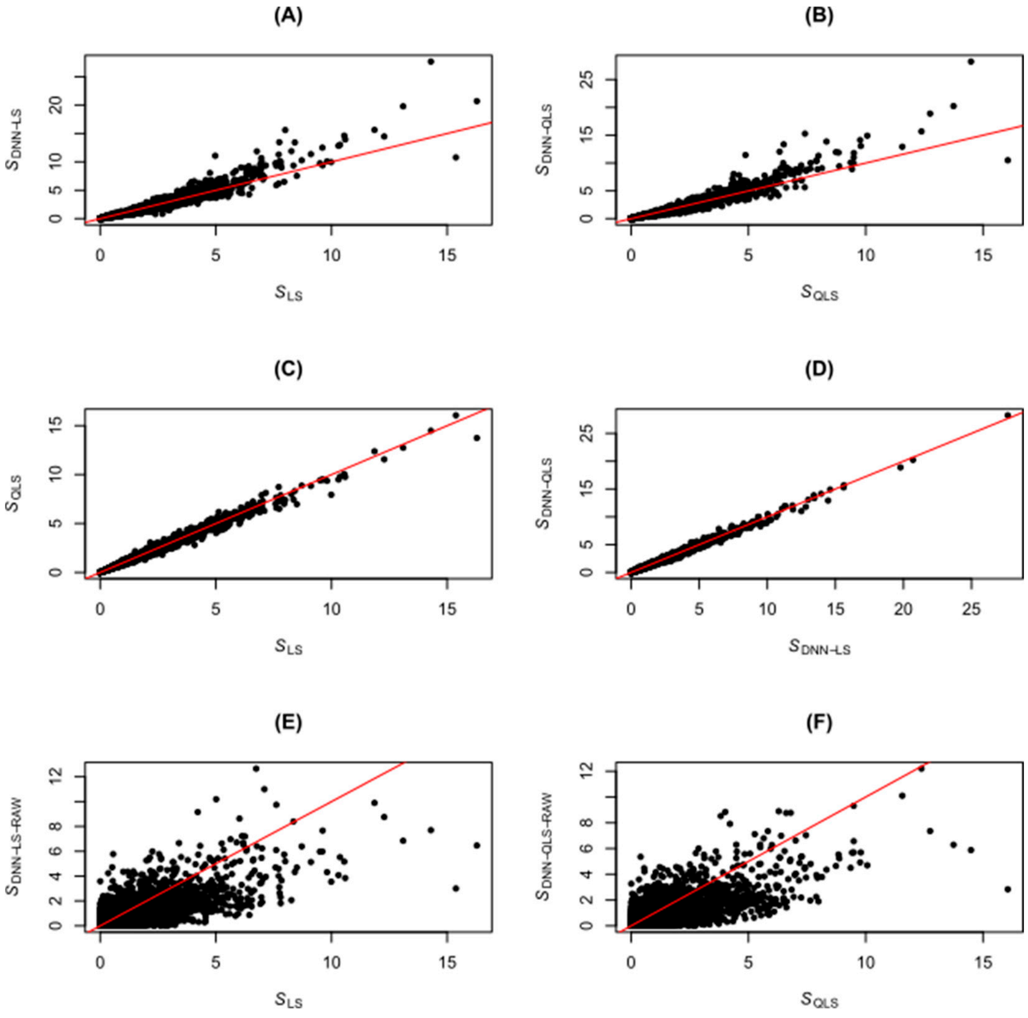

Figure S1. Consistency check for different test statistics under H_0: (A) S_{LS} VS. $S_{DNN\text{-}LS}$, (B) S_{QLS} VS. $S_{DNN\text{-}QLS}$, (C) S_{LS} VS. S_{QLS}, (D) $S_{DNN\text{-}LS}$ VS. $S_{DNN\text{-}QLS}$, (E) S_{LS} VS. $S_{DNN\text{-}LS\text{-}RAW}$, (F) S_{QLS} VS. $S_{DNN\text{-}QLS\text{-}RAW}$. The diagonal red lines represent $y = x$.

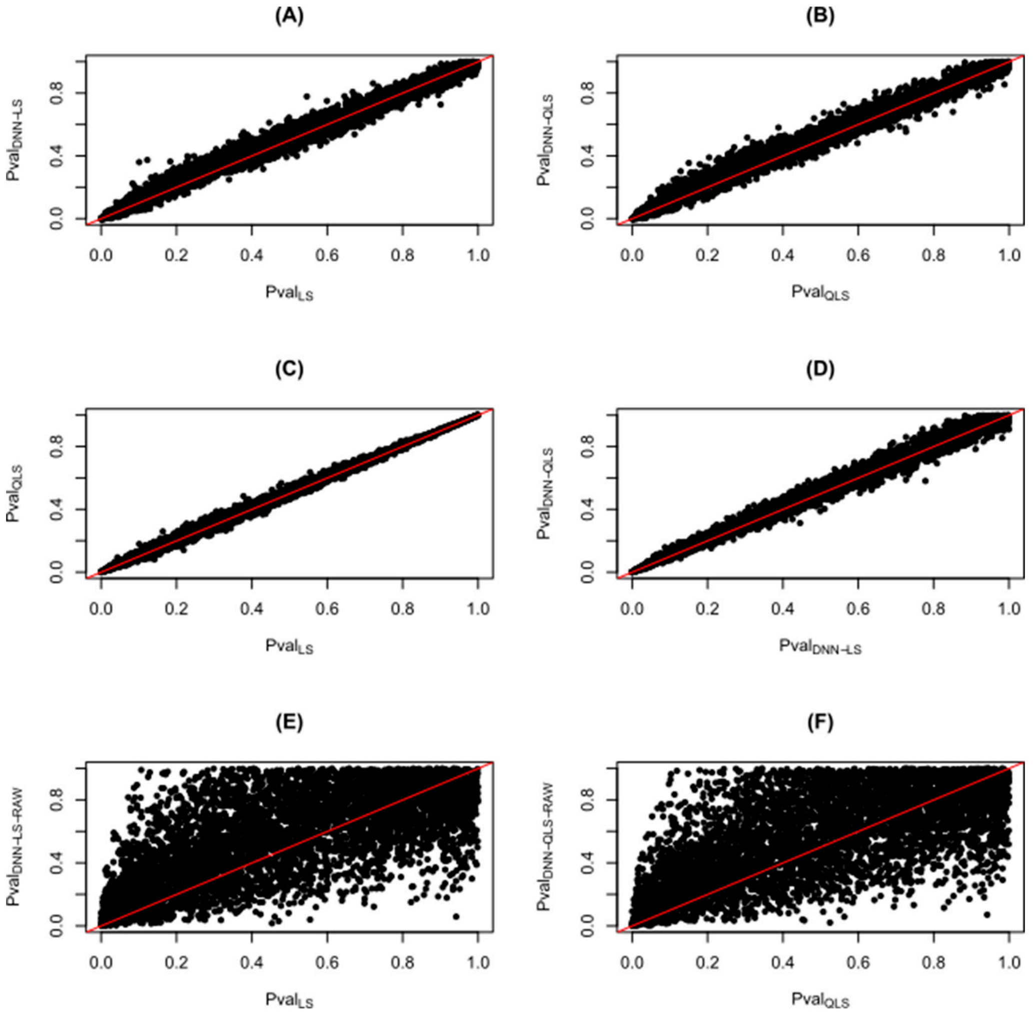

Figure S2. Consistency check for p-values under H_0 from different association tests: (A) $Pval_{LS}$ VS. $Pval_{DNN\text{-}LS}$, (B) $Pval_{QLS}$ VS. $Pval_{DNN\text{-}QLS}$, (C) $Pval_{LS}$ VS. S_{QLS}, (D) $Pval_{DNN\text{-}LS}$ VS. $Pval_{DNN\text{-}QLS}$, (E) $Pval_{LS}$ VS. $Pval_{DNN\text{-}LS\text{-}RAW}$, (F) $Pval_{QLS}$ VS. $Pval_{DNN\text{-}QLS\text{-}RAW}$. The diagonal red lines represent $y = x$.

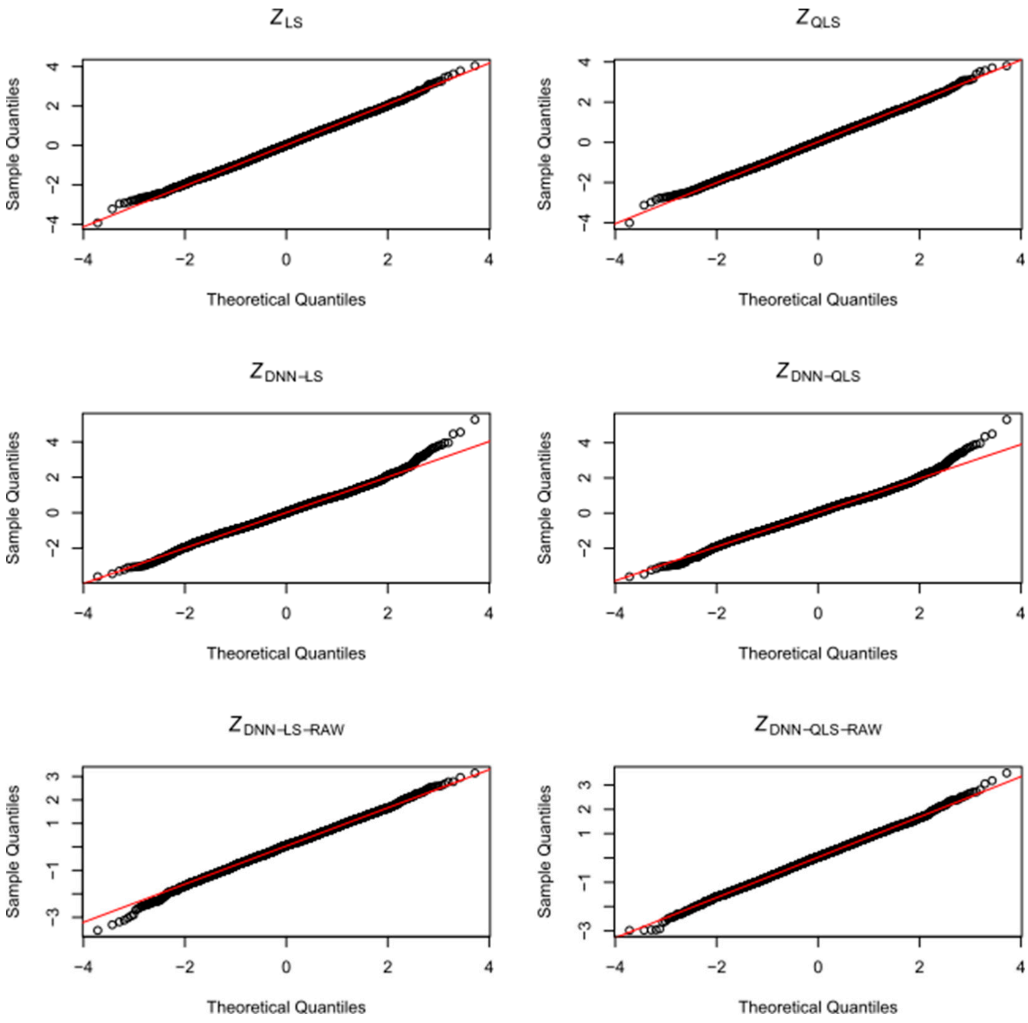

Figure S3. QQ-plots of different test statistics under H_0.

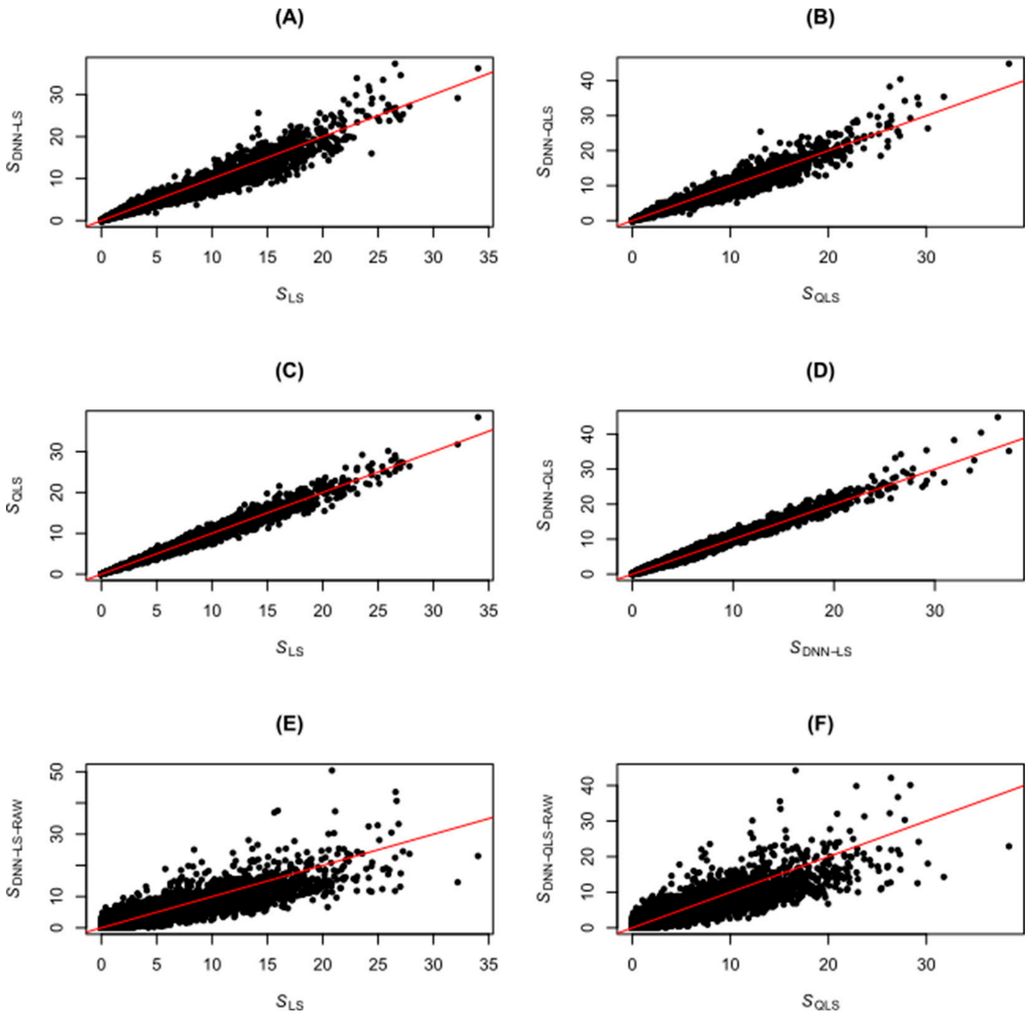

Figure S4. Consistency check for different test statistics under H_a: (A) S_{LS} VS. $S_{DNN\text{-}LS}$, (B) S_{QLS} VS. $S_{DNN\text{-}QLS}$, (C) S_{LS} VS. S_{QLS}, (D) $S_{DNN\text{-}LS}$ VS. $S_{DNN\text{-}QLS}$, (E) S_{LS} VS. $S_{DNN\text{-}LS\text{-}RAW}$, (F) S_{QLS} VS. $S_{DNN\text{-}QLS\text{-}RAW}$. The diagonal red lines represent $y = x$.

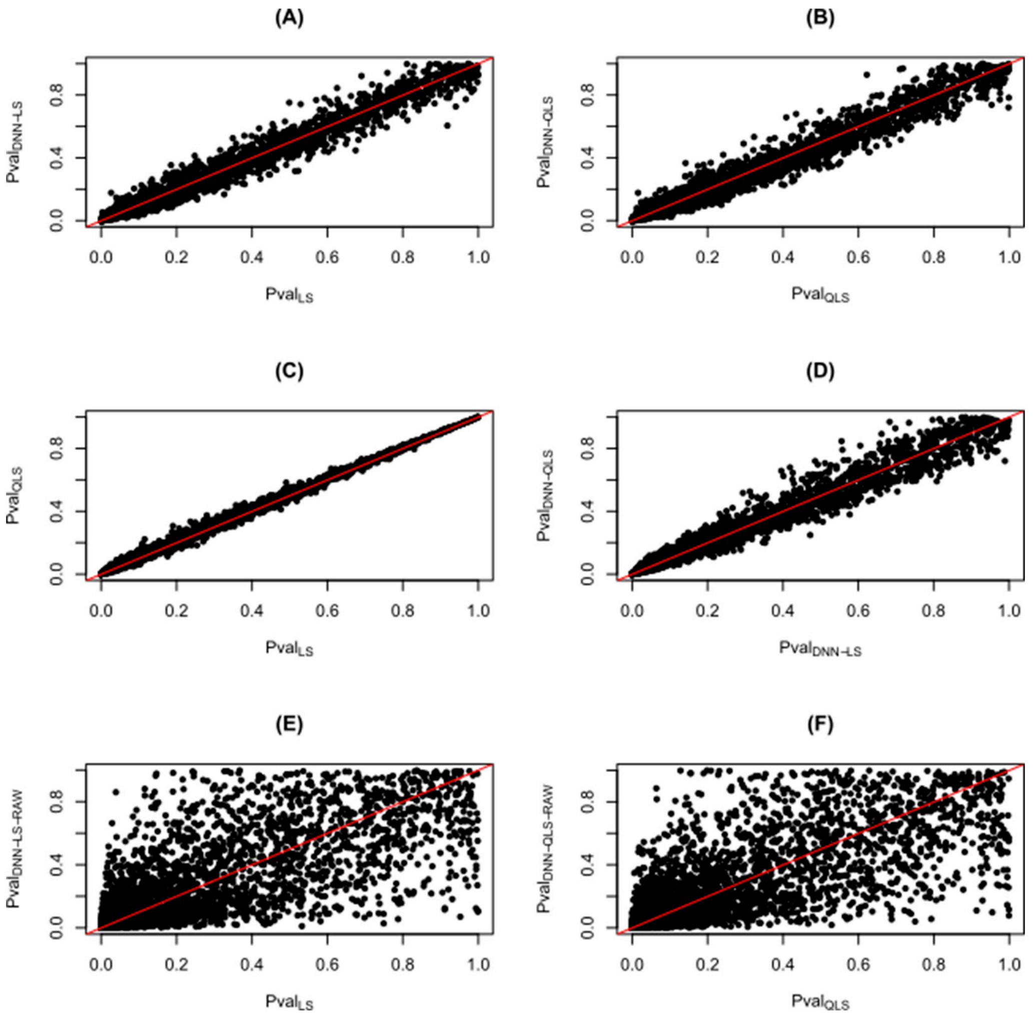

Figure S5. Consistency check for p-values under H_a from different association tests: (A) $Pval_{LS}$ VS. $Pval_{DNN\text{-}LS}$, (B) $Pval_{QLS}$ VS. $Pval_{DNN\text{-}QLS}$, (C) $Pval_{LS}$ VS. S_{QLS}, (D) $Pval_{DNN\text{-}LS}$ VS. $Pval_{DNN\text{-}QLS}$, (E) $Pval_{LS}$ VS. $Pval_{DNN\text{-}LS\text{-}RAW}$, (F) $Pval_{QLS}$ VS. $Pval_{DNN\text{-}QLS\text{-}RAW}$. The diagonal red lines represent $y = x$.

- **Supplementary tables in simulation**

Table S1. Summary of approximation accuracy under H_0 by different emulators.

	DNN-LS	DNN-QLS	DNN-LS-RAW	DNN-QLS-RAW	DNN-LS-AF	DNN-QLS-AF	DNN-ENS	DNN-ENS-AF
MAE	0.0868	0.0877	0.4144	0.4372	0.0829	0.0753	0.0840	0.0817
RMSE	0.1262	0.1285	0.5260	0.5518	0.1302	0.1192	0.1212	0.1249
R^2	0.9848	0.9839	0.7356	0.7027	0.9838	0.9861	0.9863	0.9855
Correlation	0.9926	0.9920	0.8583	0.8462	0.9922	0.9932	0.9937	0.9930

Table S2. Summary of approximation accuracy under H_a by different emulators.

		DNN-LS	DNN-QLS	DNN-LS-RAW	DNN-QLS-RAW	DNN-LS-AF	DNN-QLS-AF	DNN-ENS	DNN-ENS-AF
$k = 1$	MAE	0.1549	0.1575	0.4399	0.4416	0.1877	0.1646	0.1762	0.1846
	RMSE	0.2006	0.2084	0.5532	0.5603	0.2431	0.2253	0.2247	0.2446
	R^2	0.9927	0.9920	0.9441	0.9424	0.9892	0.9907	0.9911	0.9894
	Correlation	0.9965	0.9963	0.9720	0.9710	0.9949	0.9954	0.9959	0.9949
$k = 1.5$	MAE	0.2025	0.2230	0.4584	0.4647	0.2310	0.2091	0.1953	0.2194
	RMSE	0.2647	0.2824	0.5718	0.5794	0.3012	0.2786	0.2589	0.2904
	R^2	0.9913	0.9899	0.9595	0.9575	0.9888	0.9902	0.9919	0.9898
	Correlation	0.9964	0.9954	0.9801	0.9798	0.9946	0.9954	0.9966	0.9951
$k = 2$	MAE	0.2347	0.2325	0.4623	0.4953	0.2590	0.2503	0.2288	0.2603
	RMSE	0.3000	0.3015	0.5823	0.6246	0.3345	0.3204	0.2930	0.3325
	R^2	0.9924	0.9922	0.9713	0.9663	0.9905	0.9911	0.9929	0.9909
	Correlation	0.9964	0.9969	0.9862	0.9831	0.9957	0.9959	0.9969	0.9958
$k = 2.5$	MAE	0.2681	0.3067	0.4611	0.4841	0.3095	0.2837	0.3394	0.2926
	RMSE	0.3431	0.3848	0.5714	0.6065	0.3951	0.3606	0.4248	0.3724
	R^2	0.9924	0.9902	0.9789	0.9758	0.9899	0.9914	0.9886	0.9913
	Correlation	0.9968	0.9956	0.9900	0.9887	0.9955	0.9961	0.9965	0.9961

- **Supplementary figures in real data analysis**

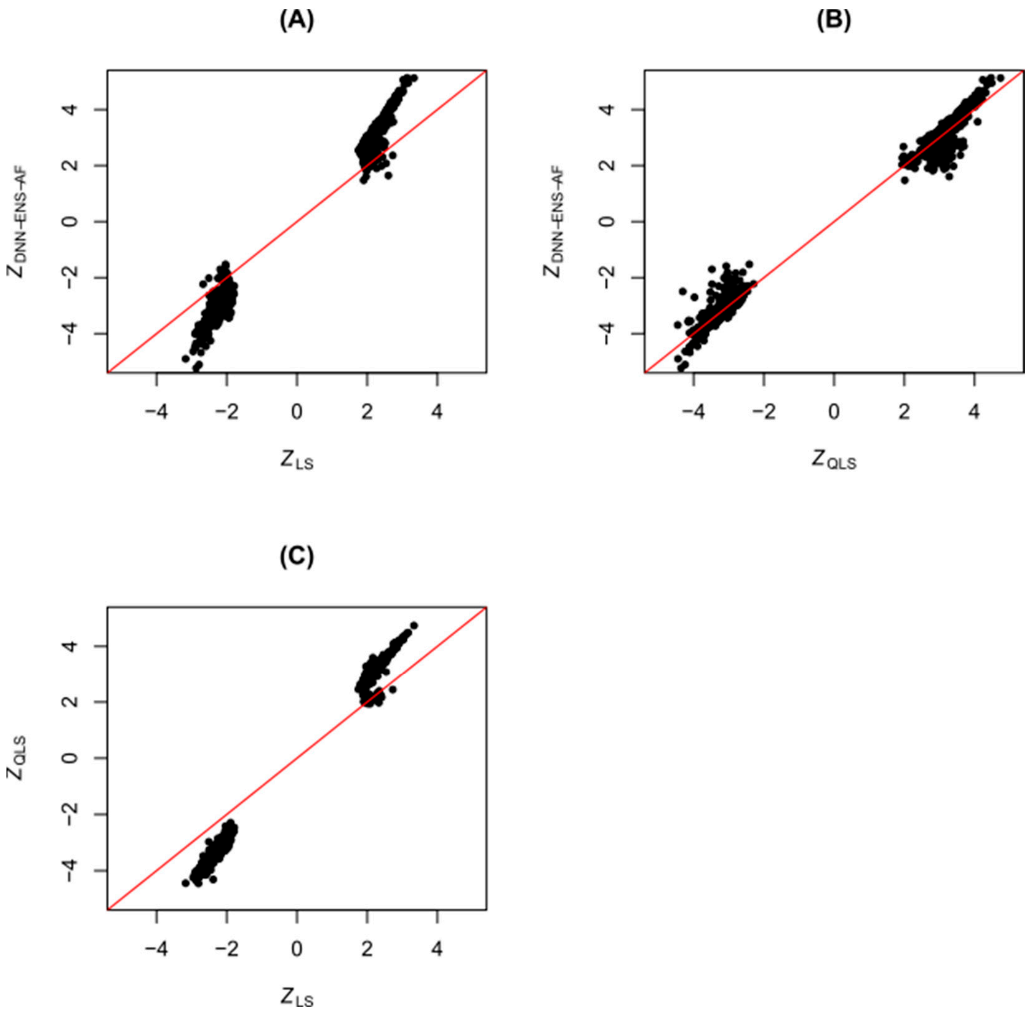

Figure S6. Scatter plot of Z-statistics of different association tests for 2547 SNPs with p-value < 0.05 in the Framingham Heart Study: (A) Z_{LS} VS. $Z_{DNN\text{-}ENS\text{-}AF}$, (B) Z_{QLS} VS. $Z_{DNN\text{-}ENS\text{-}AF}$, (C) Z_{LS} VS. Z_{QLS}. The diagonal red lines represent $y = x$.
